# Oxidative Stress: Neuropathy, Excitability, and Neurodegeneration

**DOI:** 10.1155/2019/2715326

**Published:** 2019-01-06

**Authors:** Alexandra Latini, Paula Juliana Seadi Pereira, Réjean Couture, Maria Martha Campos, Sébastien Talbot

**Affiliations:** ^1^LABOX, Departamento de Bioquímica, Universidade Federal de Santa Catarina, Florianópolis, SC 88040-900, Brazil; ^2^Département de Pharmacologie et Physiologie, Faculté de Médecine, Université de Montréal, Montréal, QC, Canada H3C 3J7; ^3^School of Health Sciences, Pontifícia Universidade Católica do Rio Grande do Sul, Porto Alegre, RS 90619-900, Brazil

The energy produced from the aerobic cellular metabolism generates free radicals. The imbalance between the production of these radicals and their elimination via antioxidant defenses leads to oxidative stress. The toxicity of these stressors contributes to protein and DNA injury, inflammation, tissue damage, and subsequent cellular apoptosis. Of note, neurons are especially susceptible to reactive oxygen species- (ROS-) mediated damages. Thus, they have a very high demand in energy, a large number of mitochondria, a limited capability to regulate glucose uptake, and weak antioxidant defenses. Alongside environmental and genetic factors, oxidative stress contributes to the onset of various pathologies such as amyotrophic lateral sclerosis, Alzheimer's disease, and Parkinson's disease, as well as diabetic-induced neuropathy and retinopathy. Thus, reduced antioxidant defenses, or the overproduction or incorporation of free radicals from the environment, can lead to neurodegeneration. This special issue on “Oxidative Stress: Neuropathy, Excitability, and Neurodegeneration” aimed at addressing the physiology, the mechanisms, and the impact of oxidative stress in neuron excitability, neurodegeneration, retinopathy, and aging. Overall, this topic compiles seven quality scientific manuscripts including three research articles and four critical reviews, which provide comprehensive evidence demonstrating the therapeutic significance of endogenous and exogenous antioxidants in the control of neurodegenerative pathologies.

Retinal detachment and blindness are increasingly more common and, while photocoagulation and anti-VEGF therapy help control the onset of the disease, these pathologies remain largely intractable. To identify novel therapeutic targets for the treatment of retinopathy of prematurity, diabetic retinopathy, and age-related macular degeneration, Chemtob and colleagues provide a state-of-the-art review addressing the role of oxidative stress and inflammatory processes in ischemic retinopathies. More specifically, they assess how microvascular change-induced ischemia promotes abnormal neovascularization and how antioxidants and anti-inflammatory agents emerged as novel therapeutic targets to reverse these ischemic retinopathies.

Kynurenic acid, one of the tryptophan metabolites, is increased in the presence of free radicals and inflammatory cytokines and was shown to have neuroactive and redox properties. Through its action on NMDA and nicotinic receptors, kynurenic acid is thought to modulate the release of neurotransmitters, and it was shown to regulate inflammation in the context of aging, neurodegeneration, and psychiatric disorders. In this issue, Chavez and colleagues reviewed various alternative mechanisms through which kynurenic acid is produced, exploring the possible involvement of the D-amino acid oxidase, indole-3-pyruvic acid, and myeloperoxidase pathways, and how such paths may be exploited to prevent neurodegeneration.

The central nervous system possesses various pathways designed to control and protect the host from the excessive production of oxidative stress, one of which is the hydrogen sulfide pathway. Impairment in hydrogen sulfide production was posited to be an early trigger for Alzheimer's, Parkinson's, and Huntington's diseases. The review article by Jung and colleagues discusses the antioxidant roles of hydrogen sulfide; how cystathionine *β*-synthase, cystathionine *γ*-lyase, cysteine aminotransferase, and 3-mercaptopyruvate sulfurtransferase enzymatic activities control its production; and how neuroinflammation might be controlled through its antioxidant capability. Among the others, hydrogen sulfide helps maintain CNS homeostasis by shielding neurons against hypoxic injury, preventing hypochlorous acid-mediated oxidative damage, enhancing glutathione generation, and repressing mitochondrial oxidative stress.

The review of Kim and colleagues explores how glutathione S-transferase superfamily of enzymes mediated cellular detoxification of oxidative damage. The omega subfamily (GSTO) is the most recently identified member of this class and appears to be expressed in a wide range of organisms including bacteria, insects, yeasts, mammals, and plants. GSTOs play an essential role in reducing the oxidative stress caused by stressors and cellular processes. Genetic polymorphisms in human GSTO1 and GSTO2 genes have been implicated in neurodegenerative diseases such as Alzheimer's and Parkinson's, and the levels of GSTO2 expression were described to be reduced in patients affected by these pathologies. Altogether, by regulating the MAPK signaling pathway, GstO1 gene helps to prevent neurotoxicity.

The uncoupling proteins (UCPs) are anion-carrier proteins found in the inner membrane of the mitochondria and are involved in the reduction of the transmembrane proton gradient. In this research paper, Antônio Silva Jr and colleagues explored whether UCP2 can protect neurons from epilepsy-induced damage. The authors demonstrated that the UCP2 mRNA expression was transiently increased in the brain post status epilepticus. UCP2 antisense oligonucleotides significantly reduced the transcript expression of UCP2 in SE-silent phase, preventing the expression of p-AKT and antiapoptotic Bcl-2 mRNA. UCP2 silencing also increases oxidative stress levels (reflected by augmented protein carbonyl levels and the activity of the antioxidant enzymes superoxide dismutase, and catalase), the liberation of proinflammatory markers, and enhanced cell apoptosis. Overall, these data revealed that UPC2 might inhibit apoptotic factors and oxidative stress in the pilocarpine model of epilepsy, and, in doing so, reduced neurodegeneration.

Given that polyphenols help to prevent neurodegeneration, Eckert and colleagues assessed how highly purified olive secoiridoids might abolish the cognitive decline typically observed in aged mice. They found that a polyphenol treatment prevented brain ATP reduction, as well as the transcript expression of NADH reductase, cytochrome c oxidase, and citrate synthase. Overall, a diet rich in purified olive polyphenols appears to protect from the decline in spatial working memory and motor coordination observed in aging. Such protective phenomena appear to be mediated by the increase in central ATP levels.

The colostrum is rich in nutrients crucial for newborn development. The latter also contains bioactive peptides, which can help to control the oxidative damage observed in neurodegenerative diseases. In this research manuscript, the group of Prof. Zabłocka presents a novel method to isolate NP-POL nonapeptide from the milk colostrum. Next, by inhibiting ROS overproduction, they found that this peptide protects PC12 cells from 6-hydroxydopamine-induced neurotoxicity. Overall, these results suggest that the NP-POL nonapeptide may help prevent ROS-triggered neurodegeneration.

